# Careful and courageous listening

**Published:** 2018-11-12

**Authors:** Marcel D’Eon

**Affiliations:** 1 University of Saskatchewan, Saskatchewan, Canada

*We are pre-empting the editorial to begin with this short essay. The regular editorial follows*.

On Saturday October 27 a gunman who later stated, “I just want to kill Jews,” opened fire in the Tree of Life synagogue in Pittsburgh killing 11 innocent, precious people. It was the worst mass murder of Jewish people in American history.^1^ The gravity of this monstrous crime against defenseless people and in many respects against all of humanity, compels us to publish this essay. We need to acknowledge those killed and how we all are affected: families and friends, the entire Jewish community of Pittsburgh - all of Pittsburgh - and all of the US, all of North America, and everyone in the world. This event has changed and will continue to change all our lives. Or it should. I am going to use the means at my disposal to respond. Each of us can do the same.

As a non-Jew, in solidarity with my Jewish friends and acquaintances, with all Jews worldwide, and with people of good will wherever they may be, I want to share three thoughts related to this unfathomable and preventable tragedy. First and foremost, kindness wins. I’ll come back to this often. Second, it is far too common that specific groups of people (immigrants, African Americans, Muslims, Jews, etc.) are targeted for violence or that violence is unleashed upon the innocent and defenseless in schools, churches, mosques, and synagogues. Finally, while tempted to point fingers and lay blame, and there is plenty to go around, let us all instead consider how we ourselves may have contributed. We are the only ones we can control. It is within our power to repair the wounds and make these senseless violent outbursts and even the slow violence of hatred less likely.

We need to ask ourselves some tough questions. How have we contributed to this situation and what can we do going forward? How inclusive and welcoming are we? Do we reach out to minorities, to people who look or behave differently from us, who come from distant lands (culturally and geographically)? Have we willingly and sincerely associated with people we do not yet know? Distancing and insulating ourselves from others is our worst response. By associating with them, we might learn that they are not very different from us; they have lives, hopes, dreams, disappointments, challenges, families, and friends pretty much like we do. If after getting to know them well they might still seem different from us. That’s when we can admit that we in fact are different from them, not only the other way around. We can do all this at a personal level and within our own spheres of influence. To make repeats of this heinous crime rare we can enact and *be* a better and a kinder way.

Beyond tending our own personal relationships with minorities, we can take a stand against those who offend. Yes, we must be that change we want to see in others. At the same time, we must also point out when people are crossing the line or moving the line or even erasing the line completely. The only thing necessary for the triumph of evil is for good people to do nothing (a modern paraphrase of Edmund Burke^2^). Can we spot the slow violent hatred and call it out? Can we resolutely, yet without venom, identify the malignancy of hate in words and actions we find on the web and in the anti-social media? Can we stand up to the bully before their momentum becomes unstoppable? Can we find the courage to articulate the values of a loving, free, and peaceful society so that the darkness is more easily exposed and disarmed? Maybe we have not been active this way as much as we should or could have. We can all do better to call out hate and live love.

Kindness wins the game of life, overall, and in the long run. The response to the hateful gun slayings in Pittsburgh and elsewhere must be strong, steady, and purposeful. No equivocation and no reluctance. We will have no short-term inaction, no aphorisms or euphemisms; let’s bring out the real force for good in the world: love. Even when others aim low, when they hurt and desert us, when they commit the unimaginable, let’s reach higher and deeper. Let’s make this planet of ours a kinder, more loving place. Out of the ruins of grief and sorrow, let’s bring forth and continue to nurture the tree of life and finish first in the only long distance race that counts.

Thank you for reading. Now I will pass you along to the planned and conventional editorial for this issue of the CMEJ – “Careful and Courageous Listening.”

It is fashionable to support the act of listening (usually from the speaker’s perspective) yet few of us take it seriously enough. We are sure that if our audience just listened better (to us) that they would see the light, our light. How few of us believe that if WE listened better WE might see it too! Whether from willful blindness (“don’t confuse me with facts”), or human hubris (“I really do know better”), or protective precaution (“I have to find some solid ground or I’ll crash”) we all tend to love our own neat and nicely arranged views of reality. They are comforting. How hard it is then to be open to new ideas and new perspectives. Critical and open-minded listening takes great courage, perhaps more than thinking or taking action. Jordan Peterson wrote, “… listening is too dangerous. The first requirement is courage, and we do not always have it.”^3^

When we as authors publish our material, the fruits of our research and our thinking, we are hoping that someone is listening, that there might be some fruitful ground upon which the seeds of change might land. We base the entire enterprise of research and public distribution of findings on the hope that people will listen even as we know deep down how hard it is (and though we might not readily admit it or even realize it, we ourselves do not listen well).

This aversion to listening persists in spite of the fact that we know we learn from listening. David Hume’s famous quote, “The truth springs from argument amongst friends”^4^ infers a deep level of listening, learning about truth, and addressing the claims and evidence presented instead of attacking the people making them (hence being and remaining friends). Argumentation is a lost art form mostly because listening is a forgotten skill.^5,6^

One way to enhance and promote listening is by truly trying to understand what the other person is saying. We do this by paraphrasing and checking that we got it right. We have used this in some of our courses for medical students.^7^ The technique was used constructively in a recent debate between Jordan Peterson and Sam Harris about the existence of God. Jordan Mamano reports:^8^

*The majority of the debate was spent with these two concerns raised; Harris critiqued Peterson for downplaying the negative consequences of religion, and Peterson probed Harris for his perceived lack of foundation to establish values and morality in the world. Brett Weinstein moderated the conversation well, asking questions to each speaker throughout to address shortcomings in their arguments. He also asked them both to steel man each other’s argument at the beginning of the second night of the debate; essentially, to put the other’s argument into their own words and make it as strong as possible (emphasis mine). This was an ingenious idea, as it served as a good recap from the first night and introduction to the second night, and warded off the potential for either Peterson or Harris to straw man their opponent and misrepresent each other’s position unintentionally for the remainder of the debate*.

The full article can be found here: https://www.thepostmillennial.com/a-recap-of-the-jordan-peterson-and-sam-harris-debate-in-vancouver/.^8^

So few of us do this because the risks are too high. Peterson again: “If you really understand a person in this way, if you are willing to enter his private world and see the way life appears to him, you run the risk of being changed yourself.”^3^ The risk on the other side is also substantial: “You’re rationalizing post-hoc. You’re matching what you want against a weak opponent so that you don’t have to change your mind. You’re propagandizing. … You’re using your conclusions to justify your proofs. You’re hiding from the truth.”^3^

Let’s hope that as we read the articles in this issue of the CMEJ we are also listening, carefully and courageously, and not protecting ourselves from the truth.

Banks and her team in “Reducing physician voiding cystourethrogram ordering in children with first febrile urinary tract infection: evaluation of a purposefully sequenced educational intervention” tackled the perennial challenge that physicians don’t always follow clinical practice guidelines. They implemented and studied a purposefully sequenced, multifaceted intervention to increase adherence to a guideline for voiding cystourethrogram use following first urinary tract infection in young children. With 109 physicians ordering 219 voiding cystourethrograms for 219 children they found, following the interventions, an increase in the monthly proportion of adherent voiding cystourethrograms ordered by pediatricians (F(2,29) = 3.38, p = .048) and non-pediatricians (F(2,28) = 14.71, p < .001).

Marlon Danilewitz et al. in “Feasibility and effectiveness of an online mindfulness meditation program for medical students” were concerned about the stubbornly high rates of psychological distress among medical students. So are we.^9^ They explored the feasibility and effectiveness of an online mindfulness intervention for medical students. Forty-five participants completed at least one of the seven modules. Mean satisfaction with the modules was 7.07±1.1 out of 10. Adherence to a regular formal meditation practice was poor. Self-compassion increased from baseline, but there was no change for burnout and empathy.

Gaboury and her team from Quebec reported on five small group interviews with 17 participants in “Strategies identified by program directors to improve adoption of the CanMEDS framework.” Francophone program directors found teaching and assessing the non-medical expert roles to be the most challenging. While this is not startling news it bears repeating and verifying in the context Canada’s francophone medical schools.

O’Brien et al. in “The effects of previous educational training on physical activity counselling and exercise prescription practices among physicians across Nova Scotia: a cross-sectional study” demonstrated the effects of previous training. Out of a population of 174 MDs they found that trained-MDs were only 22% more confident performing physical activity counselling than untrained-MDs (p<0.005). Within in-patient appointments trained-MDs and untrained-MDs had similar rates of exercise prescriptions (12%; p>0.05). The most consequential barriers were lack of time (which means other personal and institutional priorities) and perceived patient disinterest.

Kornelsen and her team in “Learners’ experiences of an enhanced surgical skills training program for family physicians” reported on the Prince Albert, Saskatchewan Enhanced Surgical Skills (ESS) program. The program appeared to meet the needs of the learners and prepared them for rural practice. Once out in practice, graduates stated that they needed mentoring and continuing education to support their growth and development.

Daniels and Daniels in “Internal medicine residents’ achievement goals and efficacy, emotions, and assessments” examined internal medicine residents’ achievement goals and how these related to their sense of self-efficacy, epistemic emotions, and valuing of formative compared to summative assessments. They found that mastery-approach goals were positively associated with self-efficacy and curiosity and negatively correlated with frustration and anxiety. Mastery-approach goals were also associated with a greater value for feedback, end-of-rotation written exams, and annual practice exams. The remaining question is then how to facilitate the acceptance of mastery-approach goals by residents. The key will be how faculty and departments organize their education programs especially competency by design.

Thompson and her team from the University of Saskatchewan in “The roles of attachment and resilience in perceived stress in medical students” examined the relationship among attachment style, perceived stress, and resilience in medical students. They used the Relationship Questionnaire and Experiences in Close Relationships Scale for attachment style. They also used the Connor-Davidson Resilience Scale and the Perceived Stress Scale. Half of their sample reported a secure attachment style. Attachment anxiety and avoidance were predictors of perceived stress. Resilience seemed to act as a partial mediator between attachment insecurity and perceived stress. These findings point to some promising areas of intervention in dealing with the widespread and debilitating experience of stress among medical student that has reached epidemic proportions.^10^

Wijeratne et al. in “Sustainability of physical exam skills in a resident-led curriculum in a large internal medicine program with competency based medical education” found that resident-led practice sessions improved OSCE performance and that peer and faculty assessments were highly correlated (but peer assigned scores were higher than those assigned by faculty). Their novel physical examination curriculum led to sustainable improvement of physical examination skills with minimal time commitment from faculty members.

Murray and his team from Manitoba (Brandon University, Manitoba Office of Rural and Northern Health, and the University of Manitoba) in “Trajectories of physicians in Manitoba, Canada: the influence of contact points of rural-focused professional learning” studied data from eight matched cohorts over the period 2004-2007 with latency periods of nine years to twelve years. They found that those physicians who were then in rural/remote practice were significantly more likely to have had additional elective experiences in rural and remote areas of Manitoba. Since this is an association and the authors only studied one factor (relevant educational experiences), we cannot be sure that the relationship is causal.

Green and Rasmussen in “Becoming a dentist: faculty perceptions of student experiences with threshold concepts in a Canadian dental program” explored, from the perspective of instructors, threshold concepts in dentistry education. From their interviews, the authors discovered four potential threshold concepts: 1) the whole patient, 2) accountability, 3) not knowing everything, and 4) problem solving during practice. Knowing these concepts may inform curriculum design.

Lafleur et al. in “Adjusting to duty hour reforms: residents’ perception of the safety climate in interdisciplinary night-float rotations” reported on a new model to accommodate residents’ duty hour reforms while maintaining safe patient care, what they call interdisciplinary night-float rotations. Using the Safety Attitudes Questionnaire, they surveyed 267 residents who had completed the novel rotation in 2015-2016. Simultaneous implementation in five hospitals of a faculty-led interdisciplinary night-float rotation proved to be logistically feasible and showed reassuring patient safety scores.

In this issue of the CMEJ, we also feature several short innovations from our new section, “You Should Try This!” Deslauriers and his team in “A physician-physiotherapist collaborative model in a family medicine teaching clinic” provided some evidence to indicate that their new model helped to provide physiotherapy services, involve physiotherapists in the training of family medicine residents, and enhanced interprofessional collaboration. They believe that this model has the potential to improve the musculoskeletal training of medical residents as well as the management of patients with musculoskeletal disorders who present to primary care facilities. Foxcroft et al. in “Implementation of a university faculty mentorship program” reported on the successful implementation of a mentorship program for faculty. Pinto et al. in “An interprofessional urban health elective focused on the social determinants of health” described the successful implementation a small inner-city community elective. Chan and his team in “Less is more: a rationalization of daily labwork” showed how integrating financial stewardship and feedback into postgraduate medical education curricula could facilitate cost-conscious patient care. Srivastava and Waghmare in “Reverse Objective Structured Clinical Examination (ROSCE)” demonstrated how the critique of clinical skills can lead to learning.

Where will these articles take us? That depends on how carefully and courageously we listen as we read. Do we find some truth in these studies and, if so, how will we respond? Will we have the courage within our roles as medical educators to do and to be different? In the larger context of recent world events and smoldering hatred, will we have the fortitude and courage to do and be different? Will we have faith that love and kindness will eventually win and be able to recognize and stop hatred whenever it rises, however disguised? Let us hope so and prepare ourselves to do so.
